# Differential Impacts of Yeasts on Feeding Behavior and Development in Larval *Drosophila suzukii* (Diptera:Drosophilidae)

**DOI:** 10.1038/s41598-019-48863-1

**Published:** 2019-09-16

**Authors:** Margaret T. Lewis, Kelly A. Hamby

**Affiliations:** Department of Entomology, University of Maryland, College Park, 20742 USA

**Keywords:** Behavioural ecology, Microbial ecology

## Abstract

Larval *Drosophila* encounter and feed on a diverse microbial community within fruit. In particular, free-living yeast microbes provide a source of dietary protein critical for development. However, successional changes to the fruit microbial community may alter host quality through impacts on relative protein content or yeast community composition. For many species of *Drosophila*, fitness benefits from yeast feeding vary between individual yeast species, indicating differences in yeast nutritional quality. To better understand these associations, we evaluated how five species of yeast impacted feeding preference and development in larval *Drosophila suzukii*. Larvae exhibited a strong attraction to the yeast *Hanseniaspora uvarum* in pairwise yeast feeding assays. However, larvae also performed most poorly on diets containing *H. uvarum*, a mismatch in preference and performance that suggests differences in yeast nutritional quality are not the primary factor driving larval feeding behavior. Together, these results demonstrate that yeast plays a critical role in *D. suzukii*’s ecology and that larvae may have developed specific yeast associations. Further inquiry, including systematic comparisons of *Drosophila* larval yeast associations more broadly, will be necessary to understand patterns of microbial resource use in larvae of *D. suzukii* and other frugivorous species.

## Introduction

Microorganisms can play a critical role in the nutritional ecology of insects and other animals^1^. Obligate symbionts, including bacteria and fungi that colonize the insect digestive tract, aid in the detoxification and digestion of phloem, wood, and other low-nutrient plant materials^1–4^. Some gut microbial symbionts also help their host synthesize essential amino acids^5^, vitamins^6,7^, or sterols^8–10^ otherwise lacking in the insect’s diet. In addition to obligate gut symbionts, insects can compensate for nutritional deficiencies within their food by supplementing their diets with free-living microbes, including bacteria, fungi, or yeast.

This latter category of nutritional interactions is particularly well documented within the genus *Drosophila* (Diptera: Drosophilidae). For many frugivorous species of *Drosophila*, yeasts provide a source of dietary protein otherwise absent from ripening fruit, a carbohydrate-rich resource^11^. While these carbohydrates are important for many aspects of adult *Drosophila* fitness, including their life span, fecundity, and survivorship^12–14^, yeast-associated protein also plays a critical role in fitness, particularly during the larval life stage. In general, *Drosophila* larvae exhibit lower survivorship in yeast-free or low yeast substrates^15–17^, and increasing the ratio of dietary protein to carbohydrates within the larval diet improves survivorship, reduces larval development time, and increases adult body mass^18–20^. Choice and no-choice behavioral studies suggest that *Drosophila* larvae preferentially feed on protein-rich food sources and will carefully regulate their food intake to consume protein quantities optimal for larval fitness^19^.

However, protein abundance within fruit and other fermenting larval substrates change over time, impacting nutritional quality for *Drosophila* larvae. During fermentation, the yeast microbial community undergoes a series of successional changes in both its species composition and density^21^. In particular, the protein to carbohydrate (P:C) ratio increases as fermentation progresses^19,22^. This microbial succession is frequently mirrored by sucessional colonization of different *Drosophila* species^21^, because individual *Drosophila* vary in their nutritional requirements^22^. Expanding our understand of the nutritional ecology of different *Drosophila* species may provide insight into larval resource partitioning and will also contribute to our knowledge of *Drosophila suzukii* Matsumura, a close relative of the model organism *Drosophila melanogaster* Meigen and a major agricultural pest in small fruit crops.

*Drosophila suzukii* is an invasive fruit fly that occupies a unique ecological niche among frugivorous *Drosophila*. Unlike other species, female *D. suzukii* possess a serrated ovipositor that enables them to lay eggs in ripening fruit^23^ during the early stages of fermentation. In contrast, most other frugivorous *Drosophila* species wait until fruit is decaying to deposit eggs. Consequentially, *D. suzukii* larvae develop under relatively protein-poor and carbohydrate-rich conditions, a nutritional niche that corresponds with larval performance in laboratory development assays. When reared on intermediate protein diets (e.g. 1:2 or 1:4 P:C ratio), larval *D. suzukii* exhibit faster development times, larger adult body sizes, and higher female ovariole numbers relative to *Drosophila biarmipes* Malloch^19^, a close relative of *D. suzukii* that colonizes decaying fruit. Furthermore, diets too rich in microbiota may have deleterious effects on larval *D. suzukii* fitness. The median lifespan of amicrobial *D. suzukii* reared on nutrient-rich sucrose-yeast diets (71 days) decreased when their natural microbiota was present (47 days). The presence of microbiota also decreased adult body size by 0.32 mg (female) and 0.11 mg (male), while slightly increasing the development period from 11.94 to 12.19 days^17^. In contrast, the microbiota/nutrient-rich diet combination does not appear to harm *D. melanogaster*; comparisons between amicrobial larvae and larvae containing their natural microbiota found no differences in larval development time^24^. These differences likely reflect adaptations by *D. suzukii* larvae to relatively nutrient-poor ripening fruit.

In addition to differences in yeast density, the composition and relative abundance of individual yeast species within a fruit changes over time. This can further impact fruit habitat suitability, as individual yeast species differentially impact larval fitness and development^16,17^. For example, *D. melanogaster* exhibit lower survivorship and smaller adult body mass when reared on diets containing the yeast *Metschnikowia pulcherrima*, compared with diets containing either *Saccharomyces cerevisiae*, *Pichia toletana*, or *Kluyveromyces lactis*^25^. Different quantities of heat-killed yeasts are needed, depending on species, to support development in larval *D. melanogaster*, suggesting that yeasts vary in their nutritional quality^20^. Indeed, the concentration and composition of key nutrients such as lipids, amino acids, mannoproteins, and fatty acids differ between yeast species^26–28^. In addition to variably impacting larval development, it is possible that these nutritional differences influence larval feeding behavior.

Larval *Drosophila* often exhibit distinct yeast feeding preferences^25,29–32^, though the level of selectivity can vary between species based on their host substrate. *Drosophila* that have a restricted host range tend to exhibit less selective feeding behavior. For example, larvae of the specialist cactophilic *Drosophila nigrospiracula*, *Drosophila mettleri*, and *Drosophila pachea* feed on yeast at the same frequency as yeast species occur within the larval substrate^33^. This behavior may indicate that larvae with a restricted host range cannot afford to evolve specialized microbe feeding behaviors, as microbial communities are ephemeral and often vary between conspecific host substrates^33^. In contrast, generalist *Drosophila* such as *Drosophila mojavensis* or *D. melanogaster* exhibit distinctive yeast feeding preferences in both field and laboratory settings^30,33^.

This selective foraging behavior may reflect perceived differences in yeast resource quality. The larval chemosensory system contains an array of gustatory and olfactory neural receptors^34^ that allow larvae to discriminate between food sources based on nutritional factors such as the identity and availability of sugars and amino acids^35–37^. Therefore, larval *Drosophila* may selectively feed on yeasts that best support their fitness, with specific *Drosophila* – yeast associations dependent on the fruit microbial community and stage of fruit decay typically encountered.

Previous field surveys indicate that *D. suzukii* larvae feed upon a distinct yeast fauna, with one species of yeast, *Hanseniaspora uvarum*, predominating in the gut^38,39^. *Hanseniaspora uvarum* is a widespread yeast species that occurs at high frequency in the early stages of fruit fermentation^21,40^ and can be antagonistic to other species of fungi, including yeast^41^. Therefore, larval feeding patterns may reflect *H. uvarum*’s abundant field density; alternatively, these patterns may indicate feeding preferences.

To better understand the nature of these interactions, we evaluated larval *D. suzukii* feeding preference and performance in response to diets prepared using five different species of yeast, including the model organism *S. cerevisiae* and natural yeast associates of larval *D. suzukii*^38^. We hypothesized that larvae would exhibit a significant preference for the yeast that best supported their fitness. While larvae did exhibit a strong preference for *H. uvarum* in laboratory preference assays, this preference negatively correlated with performance. Our results suggest larval *D. suzukii* yeast feeding preferences may be driven by factors beyond nutritional quality.

## Results

### Larval development assays

We evaluated how three species of yeasts isolated from field collected *D. suzukii* larvae (*H. uvarum*, *Pichia kluyveri*, and *Issatchenkia terricola*) and diets without yeast (negative control) impacted fitness and development in larval *D. suzukii* (Supplementary Table S1). As a positive control, we also prepared diets using commerical *Saccharomyces cerevisiae*, because this species is frequently used as a model organism to study *Drosophila* – yeast interactions. To ensure the diet microbial community remained static and to remove confounding effects due to yeast growth rate, development assays were conducted with standardized amounts of frozen yeast and diets were autoclave sterilized to heat-kill all microbes. Diet treatments were monitored daily for both pupation and adult eclosion, and emergence data were used to calculate larval (1^st^ instar larvae to pupa) survivorship and development time, pupal (pupa to adult) survivorship and development time, and total (1^st^ instar larvae to adult) survivorship and development time. Thorax and wing length measurements were also taken to quantify the body size of any emerged adults. Diets were prepared on three separate occasions (N = 3) with 6 dishes per treatment for which subsamples were averaged prior to analysis.

#### Survivorship

**Figure 1:**
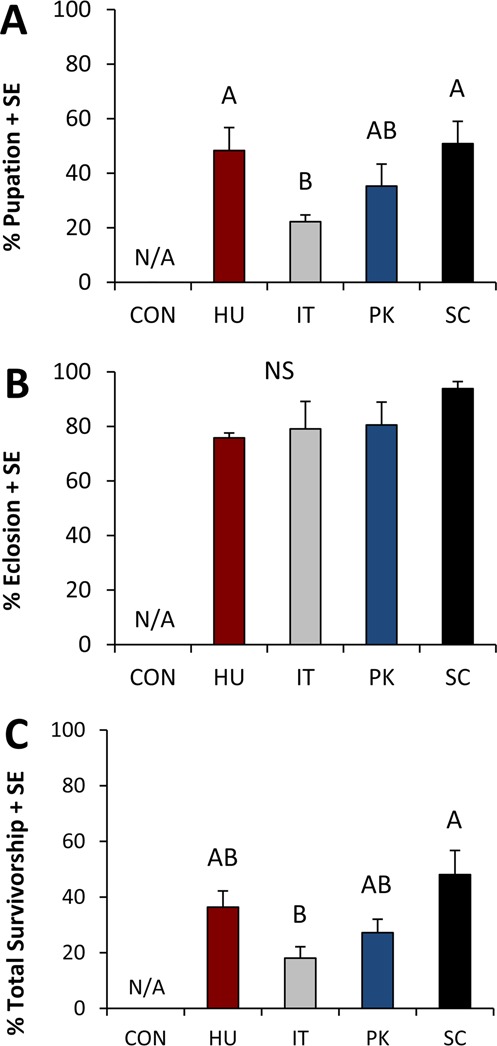
*D. suzukii* survivorship in response to experimental diets. Larvae were reared on diets containing either no yeast (CON), *Hanseniaspora uvarum* (HU), *Issatchenkia terricola* (IT), *Pichia kluyveri* (PK), or *Saccharomyces cerevisiae* (SC). Mean percent survivorship + standard error of (**A**) larvae that pupated (larval survivorship), (**B**) pupa that eclosed as adults (pupal survivorship) and (**C**) larvae that successfully eclosed as adults (total survivorship) (N = 3 replicate experiments) are presented. Data were analyzed using a linear mixed model. Yeast species significantly impacted the larval (*F*_3,6_ = 6.688, *P* = 0.024) and total (*F*_3,6_ = 6.466, *P* = 0.026) survivorship but not pupal survivorship (*F*_3,6_ = 1.857, *P* = 0.238). Within a graph, bars that do not share a letter are significantly different (p < 0.05). Control larvae were excluded from all analysis due to 0% survival.

Individual yeast species significantly affected larval survivorship (1^st^ instar larvae to pupa; *F*_*3*,*6*_ = 6.688, *P* = 0.024). Larvae reared on a yeast-free diet (negative control) exhibited 0% survivorship across all replicates (Fig. 1), indicating that yeast is essential for *D. suzukii* development. Apart from the yeast-free control, the lowest rates of larval survivorship occurred on diets containing *I. terricola* (22.2 ± 2.5%; mean ± standard error), while there was higher larval survivorship on either *S. cerevisiae* (50.8 ± 8.2%) or *H. uvarum* (48.3 ± 8.4%) (Fig. 1A). Total survivorship patterns were similar (Fig. 1C; *F*_*3*,*6*_ = 6.466, *P* = 0.026), with the highest percentage of larvae sucessfully emerging as adults in response to either *S. cerevisiae* (48.1 ± 8.7%) or *H. uvarum* (36.4 ± 5.8%). In contrast, pupal survivorship (pupa to adult; *F*_*3*,*6*_ = 1.857, *P* = 0.238) was not impacted by diet treatments.

#### Development time

**Figure 2:**
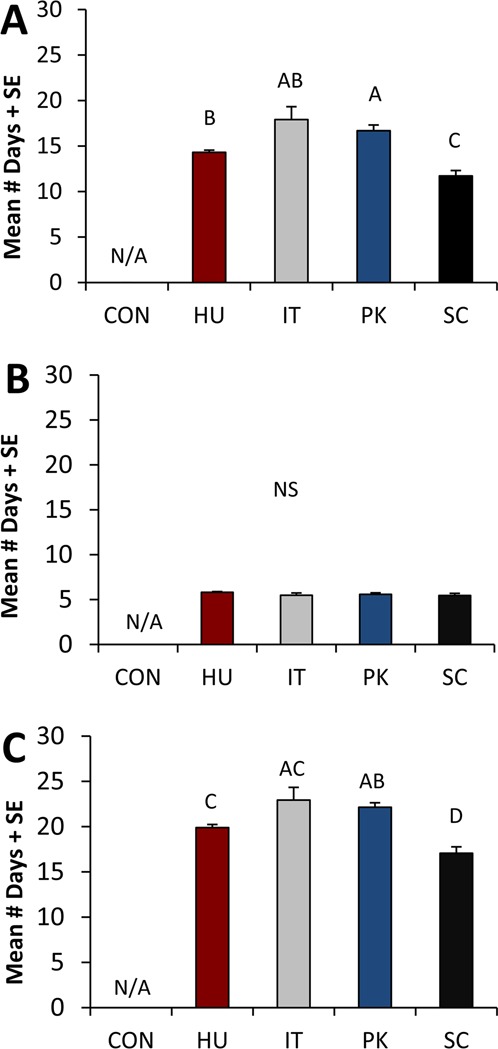
*D. suzukii* development period in response to experimental diets. Larvae were reared on diets containing either no yeast (CON), *Hanseniaspora uvarum* (HU), *Issatchenkia terricola* (IT), *Pichia kluyveri* (PK), or *Saccharomyces cerevisiae* (SC). Mean number of days for development + standard error for (**A**) larval development (1^st^ instar to pupa), (**B**) pupal development (pupa to adult) and (**C**) total development (1^st^ instar to adult) (N = 3 replicate experiments) are presented. Larval and adult development data were analyzed using a mixed-model ANOVA with a (treatment residual variance)^−1^ weighting factor. Pupal development times were analyzed without a weighting factor. Within a graph, bars that do not share a letter are significantly different (P  < 0.05).

The fastest larval development period occurred on diets containing *S. cerevisiae* (1^st^ instar larvae to pupa; *F*_3,8_ = 13.418, *P* = 0.002), with larvae taking 11.7 ± 0.6 days to pupate. Larvae reared on *H. uvarum* took 14.3 ± 0.2 days to reach the pupal stage, and the slowest larval development times occurred in response to either *I. terricola* or *P. kluyveri* (Fig. 2A, Supplementary Fig. S1A). Patterns in the total development times were similar (Fig. 2C; *F*_3,8_ = 12.799, *P* = 0.002), with the fastest larval to adult development times again occurring in response to *S. cerevisiae* (see also Supplementary Fig. S1B). We observed no significant differences in pupal development time among treatments (*F*_3,6_ = 3.369, *P* = 0.096; Fig. 2B).

#### Adult body size

The individual yeast diets significantly affected thorax length in both male (*F*_3,6_ = 9.880, *P* = 0.010) and female (*F*_3,8_ = 20.467, *P* < 0.001) *D. suzukii* (Table 1). The largest thorax lengths were observed in male (1.08 ± 0.01 mm) and female (1.24 ± 0.01 mm) flies reared on *S. cerevisiae*. In contrast, the smallest flies observed were those reared on a *H. uvarum* diet. Flies reared on either an *I. terricola* or a *P. kluyveri* based diet also exhibited a reduced body size relative to *S. cerevisiae* and were slightly larger than those observed from *H. uvarum*. Similar patterns emerged in the wing length of both male (*F*_3, 6_= 6.983, *P* = 0.022) and female (*F*_3,8_ = 17.462, *P* < 0.001) *D. suzukii*, with the largest wings occuring in flies reared on *S. cerevisiae* and the smallest wings in flies reared on *H. uvarum* (Table 1).

**Table 1:** Thorax and wing lengths of male and female *D. suzukii* reared on experimental diets.

Sex	Treatment	Total # Flies Measured	Average Wing Length (mm) ± SE	Average Thorax Length (mm) ± SE
Female	*H. uvarum*	105	2.14 ± 0.04 C	1.09 ± 0.02 C
	*I. terricola*	39	2.26 ± 0.01 B	1.17 ± 0.01 B
	*P. kluyveri*	61	2.21 ± 0.08 ABC	1.14 ± 0.05 ABC
	*S. cerevisiae*	100	2.35 ± 0.01 A	1.24 ± 0.01 A
Male	*H. uvarum*	24	1.83 ± 0.05 b	0.90 ± 0.03 b
	*I. terricola*	28	1.99 ± 0.02 ab	1.00 ± 0.01 ab
	*P. kluyveri*	36	1.96 ± 0.07 ab	1.00 ± 0.03 ab
	*S. cerevisiae*	72	2.06 ± 0.01 a	1.08 ± 0.01 a

Wing and thorax length measurements were taken for adult *D. suzukii* that successfully emerged; therefore, the total number of flies measured within a given treatment varied. These subsamples were averaged by sex within each trial prior to analysis, and the mean wing length (millimeters) ± standard error (N = 3) is presented. Statistical analyses were conducted separately for male and female flies and for wing length and thorax length. Within a given sex and measurement, values that do not share a letter are statistically different (P < 0.05).

#### Diet nutritional analysis

To compare nutritional content between our experimental diets and the standard *Drosophila* diet used to maintain our laboratory stocks, we conducted proximate nutritional analysis on all diets used in this study and a standard diet prepared using freeze-dried *S. cerevisiae* (Supplementary Table S1). Nutrient analyses were repeated twice, using diets prepared on two separate dates.

We observed no major nutritional differences between any of our experimental diets, which were all prepared using yeast cells scraped from media plates. However, diets prepared without yeast (negative control) consistently had the lowest caloric content and fell below the detectable protein threshold in both replicates. In contrast, diets prepared using freeze-dried *S. cerevisiae* had higher caloric values (52.5 ± 0.5 calories per 100 grams diets; N = 2 replicates) relative to any other treatment. For example, diets prepared using wet *H. uvarum* cultures had the second highest caloric value at 43.0 ± 1.0 calories per 100 grams. We also observed higher relative amounts of ash (inorganic residue), carbohydrates, and protein within diets prepared using freeze-dried yeast cultures (Supplementary Table S2).

### Larval yeast preference

We evaluated larval *D. suzukii* feeding preference for five species of yeast (*H. uvarum*, *P. kluyveri*, *I. terricola*, *W. pijperi*, and *S. cerevisiae*) through two-choice feeding assays. In each assay, larvae were placed on a large water-agar plate provisioned with two yeast options (colored red or blue) on opposite ends of the plate, and larval feeding preferences were assessed after one hour based on the color of the alimentary canal. For each set of two-choice tests, we conducted 12 replicate assays.

**Figure 3:**
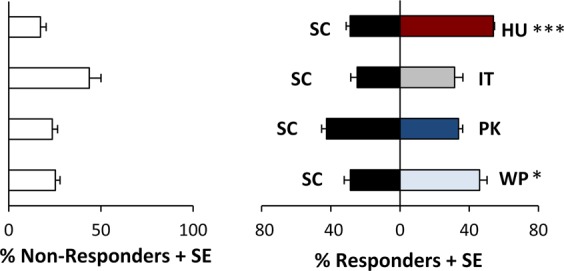
Larval *D. suzukii* feeding preferences for all comparisons involving *S. cerevisiae*. Mean percentage of larvae + standard error that responded to each yeast paired with the mean percentage of larvae + standard error that did not respond (N = 12 replicate binary choice assays) are presented for HU = *Hanseniaspora uvarum*, IT = *Issatchenkia terricola*, PK = *Pichia kluyveri*, SC = *Saccharomyces cerevisiae*, WP = *Wickerhamomyces pijperi*. Responding larvae were analyzed using a paired t-test, with larvae demonstrating significant preference for one yeast over another at the *P < 0.05, **P < 0.01, or ***P < 0.001 level.

Overall, *D. suzukii* larvae preferred *H. uvarum* (*T*_11_ = 7.214, *P* < 0.001) and *W. pijperi* (*T*_11_ = 2.286, *P* = 0.043) over *S. cerevisiae* (Fig. 3). For example, 54.0% ± 2.3% (mean ± standard error) of the larvae assayed chose to feed on *H. uvarum*, compared with the 28.8% ± 2.3% that chose to feed on *S. cerevisiae*. Similarly, in comparisons between *W. pijperi* and *S. cerevisiae*, 46.0% ± 4.3% and 28.7% ± 3.6% of larvae assayed chose to feed on each yeast respectively (Fig. 3). However, larvae exhibited no significant feeding preferences in pairwise comparisons between *S. cerevisiae* and either *P. kluyveri* or *I. terricola*. Larvae also demonstrated no significant feeding preferences in pairwise comparisons of *I. terricola*, *P. kluyveri*, and *W. pijperi* (Supplementary Tables S3–S5).

**Figure 4:**
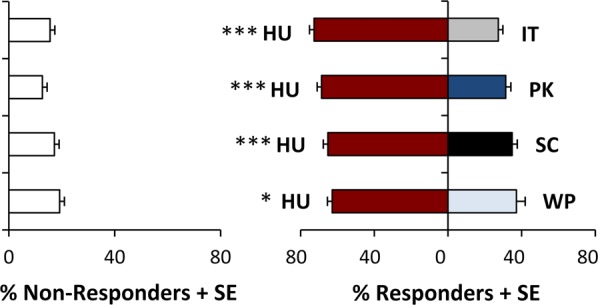
Larval *D. suzukii* feeding preferences for all comparisons involving *H. uvarum*. Mean percentage of larvae + standard error that responded to each yeast paired with the mean percentage of larvae + standard error that did not respond (N = 12 replicate binary choice assays) are presented for HU = *Hanseniaspora uvarum*, IT = *Issatchenkia terricola*, PK = *Pichia kluyveri*, SC = *Saccharomyces cerevisiae*, WP = *Wickerhamomyces pijperi*. Responding larvae were analyzed using a paired t-test, with larvae demonstrating significant preference for one yeast over another at the *P < 0.05, **P < 0.01, or ***P < 0.001 level.

Across all binary comparisons of larval feeding preference, *H. uvarum* elicited the strongest feeding response in *D. suzukii* (Fig. 4). In addition to demonstrating significant preferences for *H. uvarum* over *S. cerevisiae*, significantly more larvae chose to feed on *H. uvarum* over *P. kluyveri* (*T*_11_ = 7.468, *P < *0.001), *I. terricola* (*T*_11_ = 8.601, *P* < 0.001), and *W. pijperi* (*T*_11_ = 3.042, *P* = 0.011).

## Discussion

*Drosophila suzukii* encounters a diverse microbial community within fruit that undergoes successional changes in both density and species composition^21,42,43^, allowing them to selectively feed on a wide variety of yeast microbes that play a critical role in their life history. Larvae exhibit a strong attraction to live yeast cultures, and yeasts are important components of *D. suzukii*’s diet. Similar to previous work^16,17^, we found that larvae reared in a completely yeast-free environment universally failed to pupate or eclose. Our ability to rescue larval development with heat-killed microbes confirms that yeasts provide *D. suzukii* larvae with a source of protein and essential nutrients not otherwise found in fruit or fly diets^10,44,45^. We initially hypothesized that larvae would preferentially feed on certain species of yeast based on perceived differences in resource quality. However, the mismatch between larval yeast preference and performance suggests that larvae do not discriminate between yeast species based on nutritional quality alone and instead there may be alternative mechanisms shaping *D. suzukii*’s yeast associations.

Results from this study suggest that *D. suzukii* larvae have developed a close association with *H. uvarum*. In binary laboratory choice assays, *D. suzukii* larvae preferentially fed on *H. uvarum* over alternative natural yeast associates as well as *S. cerevisiae*. This result is consistent with previous reports that *H. uvarum* predominates the culturable larval gut microbial community in geographically distant populations of *D. suzukii*^38,39^ and to the best of our knowledge, is the first evidence that larval *D. suzukii* exhibit feeding preferences for specific yeast species. Our studies suggest that larval feeding is not random; despite being confined to a single fruit throughout development, larval *D. suzukii* appear to deliberately seek out and feed on *H. uvarum*, indicating that there may be an association between these two organisms.

We initially hypothesized that *D. suzukii* preferentially fed on *H. uvarum* because it was a higher quality yeast that better supported larvae. Instead, larvae exhibited reduced performance on diets containing *H. uvarum*. Larvae reared on *H. uvarum* developed more slowly relative to diets containing *S. cerevisiae*, and adult body size was smaller compared to individuals reared on diets prepared with *S. cerevisiae*, *P. kluyveri*, or *I. terricola*. Surprisingly, the most robust fitness occurred on diets containing *S. cerevisiae*, with higher rates of surviorship and shorter developmental times relative to flies reared on diets containing natural yeast associates. Flies reared on *S. cerevisiae* based diets also had significantly larger adult body sizes, a trait that generally indicates higher levels of fecundity, survival, and mating success^46^. This higher performance on *S. cerevisiae* was surprising, because *Drosophila* rarely associate with this species of yeast in nature^38,39,47^ and larvae did not prefer *S. cerevisiae* in binary choice assays. It is possible that these results reflect phenotypic plasticity in resource use, with larvae able to exploit and perform well on diverse yeast resources despite specialization towards *H. uvarum*^48^. Similar plastic behavior may occur in adult *D. suzukii* during winter and early spring when ripe fruit is scarce in temperate climates; under no-choice laboratory conditions female *D. suzukii* will accept and oviposit into less optimal resources including mushroom, apple, and chicken-manure based diets^49^. The enhanced performance we observed could also result from laboratory colony selection effects. All flies used in this study came from a *D. suzukii* colony reared for over 50 generations on a standard *S. cerevisiae* based diet. Alternatively, the benefits conferred from *S. cerevisiae* may reflect commercial selection effects, because the particular strain of yeast used in this study was originally selected for making bread, which could impact protein content and secondary metabolite production. Since *D. suzukii* larvae do not frequently encounter *S. cerevisiae* in nature, they may not have recognized this particular yeast as a superior food source.

This mismatch in larval yeast preference and performance suggests that *D. suzukii* larvae do not discriminate between individual species of yeast based solely on the nutritional quality of the yeast. Yeast quantity, rather than quality, may be a more important determinant of larval fitness. In this study, *D. suzukii* larvae developed under protein-limited conditions. We prepared our fly diets following the standard recipe used to maintain our laboratory *Drosophila* stocks, changing only the species of yeast added. Instead of using dehydrated yeast, we harvested and weighed all our yeast directly from PDA plates, a step that likely reduced the nutritional value relative to the original, freeze-dried yeast recipe (Table S2). The protein content in our freeze-dried yeast diet averaged 2.42%, similar to protein concentrations (1.11% and 2.0%) in other published *Drosophila* diets^11,17^. In contrast, average protein concentration in our experimental diets ranged from 0.78–1.24%. Negative fitness impacts due to the limited protein conditions within our diets were likely compounded by the use of heat-killed microbes. By steam sterilizing our diets to avoid differences due to variable yeast growth rates^50^ and contamination by other microbes, we stopped microbial growth. This may have created yeast shortages typically not observed in the field where natural yeast growth or larval niche construction and yeast seeding would increase yeast abundance over time^51,52^.

Yeast shortages and the associated low dietary protein may have detrimentally impacted larval fitness, especially prolonging development. In this study, the fastest development time occurred in larvae reared on *S. cerevisiae*, with first instar larva to pupa development taking an average of 11.7 ± 0.6 days and first instar larva to adult devleopment taking an average of 16.9 ± 0.8 days. In contrast, other studies have observed that *D. suzukii* development times on *S. cerevisiae*-based fly diets takes 6.0–7.1 days for pupation^16,53^ and 11.9–12.8 days for adult emergence^17,53^ at temperature levels comparable to our study conditions^16,17,53^. Beyond development time, low protein conditions can negatively impact other aspects of larval fitness, including survivorship and adult body size^18–20^. Within carbohydrate-rich ripening fruit^11^, larval *D. suzukii* likely rely on the yeast microbial community to obtain sufficient protein for development.

Given the importance of dietary protein for development^17^, *D. suzukii* larvae may prioritize feeding on yeasts that are abundant and readily available over selectively seeking higher quality species. For example, *D. suzukii* larvae may preferentially feed on *H. uvarum* because it predominates yeast microbial communities during the early stages of fermentation, thus providing a more abundant source of protein. Previous studies have demonstrated that *D. suzukii* larvae provisioned with live cultures of *H. uvarum* generally experience a robust fitness phenotype relative to other yeast associates^16,17^. The higher performance observed in these studies likely reflects a higher yeast abundance when using live cultures, because *Drosophila* spp. larval feeding increases yeast abundance within their host substrates in field^39^ and laboratory experiments^52^, and live yeasts are able to continually grow during development assays. Microbial abundance positively correlates with larval growth rates in *D. melanogaster*, suggesting that a microbe’s ability to proliferate may be one of the most important predictors of its effect on larval fitness^20^. Therefore, *D. suzukii* larvae may benefit from *H. uvarum*’s widespread and competitive nature^21,40^, as it means that *H. uvarum* can quickly increase its density, providing larvae with a consistent and abundant source of protein.

Beyond its ability to proliferate, there are a number of other factors that could mediate larval attraction to *H. uvarum*. For example, *H. uvarum*’s attractiveness may reflect yeast adaptations that enhance its fitness. Adult *Drosophila* disperse yeasts^54,55^, and more attractive yeast strains experience higher rates of dispersal^56^. Larval feeding may also confer competitive advantages to yeast by promoting yeast growth or genetic diversity^51,52,57^. Alternatively, some strains of *H. uvarum* produce “killer toxins” that may help larvae outcompete harmful plant pathogenic fungi^58^ or create an enemy-free space^59^, consequentially enhancing larval fitness through measures not quantified in this study. A similar competitive advantage has been proposed for *D. melanogaster*, with larvae parasitized by the wasp *Asobara tabida* preferentially feeding on yeast species that enhance their ability to melanotically encapsulate parasitic attacks^60^. It is also possible that *H. uvarum* confers additional fitness benefits during the adult life stage not quantified in this study such as adult survivorship^61^, adult cuticular pheromone production^61^, or reproductive outputs such as ovariole numbers^19^.

*Hanseniaspora uvarum* is a widespread yeast species frequently isolated from fermenting fruits and insects^40^, including adult *Drosophila*. A survey of *Drosophila* spp. yeast associations found that with a few exceptions, the *H. uvarum* species complex was the most abundant OTU isolated from the gut of adult flies^47^, suggesting a general feeding association between *Drosophila* and *H. uvarum*. Volatiles associated with *H. uvarum* are also highly attractive to multiple adult species, including *D. suzukii* and *D. melanogaster*^62,63^. While further work is necessary to fully understand the mechanism and nature of *H. uvarum*’s association with *D. suzukii*, it is clear that *H. uvarum* strongly impacts *D. suzukii*’s ecology, similar to other *Drosophila*.

The extent to which adult yeast associations overlap with the larval life-stage remains unclear. Adult flies are highly mobile insects, capable of visiting a diverse community of host plants, which provides them a different, broader feeding niche than larvae^64^. Field and laboratory surveys of cactophilic *Drosophila* yeast associations report differences between adult and larval yeast preferences^29^. For example, in laboratory assays, female *Drosophila buzzati* exhibited a significant preference for ovipositing and feeding on cactus inoculated with *Pichia cactophila* relative to *Clavispora opuntiae*^65^, while larvae exhibited high attraction to both yeast species^66^. In addition, surveys of adult feeding behavior on decaying oranges found that *Drosophila* spp., including *D. melanogaster* and *D. pseudoobscura*, fed more frequently on yeasts available at the surface of necrotic tissue compared to yeasts colonizing the interior fruit rot, suggesting a spatial separation between adult and larval feeding niches^67^.

Yeast associations and preferences have been fairly well surveyed within adult frugivorous *Drosophila*^47,68–71^. However, records of natural yeast associations within frugivorous larvae are more limited. Previous laboratory studies using *D. melanogaster* and cactophilic *Drosophila* larvae demonstrate that larvae have specific yeast preferences^25,29,32^, and these preferences vary between species. In pairwise yeast preference comparisons, *D. buzzati* and *Drosophila aldrichi* exhibited slight differences in their yeast preferences^66^. Also, within decaying oranges, *D. arizonensi* and *D. melanogaster* consumed *H. uvarum* at lower frequencies than it occurred in the orange microbial community^33^, a result that suggests larvae were avoiding *H. uvarum*, in contrast to the strong preference for *H. uvarum* we observed in *D. suzukii*. Within fermenting fruit, it therefore seems plausible that different species of *Drosophila* larvae develop different yeast preferences and associations, and that these associations shift across temporal niches within fermenting fruit. For example, *D. suzukii* larvae could develop closer associations with early stage fermentation communities compared to *D. melanogaster* and other late stage colonizers. Systematic comparisons of larval yeast preferences and surveys of larval yeast associations would be needed to test this hypothesis.

### Conclusions

Because yeasts play such a critical role in *D. suzukii*’s ecology, there may be opportunities to exploit these interactions for more sustainable pest management^72^. Yeast associated volatiles could be integrated into monitoring programs for *D. suzukii*. Fermentation based-lures have already been developed, but current trapping systems remain difficult to use due to issues with trap selectivity and poor correlations between adult trap captures and larval infestation^73,74^. It may be possible to use yeast volatile components specifically attractive to *D. suzukii*^63^ to develop a more selective trapping system. Similarly, yeast-associated volatiles could also be incorporated into a push-pull system for *D. suzukii*^75^.

Recent research efforts have also focused on incorporating yeasts into feeding baits or biopesticides specific to *D. suzukii*. In laboratory trials, adult and larval *D. suzukii* exhibited reduced fitness after ingesting *S. cerevisiae* that was genetically modified to express double-stranded RNA^76^. Yeasts have also been tested as potential phagostimulants for insecticide applications, with variable efficacy. Adding yeast to either spinosad or cyanotraniliprole increased adult mortality and decreased larval infestation compared to treating with the insecticide alone^77^. However, efficacy varied between yeast species and insecticides, with highest efficacy observed when using *S. cerevisiae* and commercial formulations of the yeast *Aureobasidium pullulans* as phagostimulants^77^. Similarly, laboratory assays also reported that combinations of spinosad and *H. uvarum* increased *D. suzukii* mortality relative to the insecticide alone^78^. In contrast to these studies, recent field and laboratory assessments found that adding *S. cerevisiae* to various organic insecticides did not improve control of *D. suzukii* in either semi-field or laboratory assays, a difference that may reflect variation in *D. suzukii*’s physiological status between studies^79^.

There appears to be considerable variation in how *D. suzukii* interacts with yeasts throughout its life history. Both adult and larval *D. suzukii* exhibit specific yeast preferences, and during the adult life stage, different species of *Drosophila* vary in their response to specific yeast volatile components^63^. Furthermore, the physiological status of adult flies can also influence behavioral responses. For example, unmated or reproductively immature females exhibit a higher attraction towards yeast volatiles^80,81^, and winter and summer morph *D. suzukii* vary in their responses to fungal-associated volatiles^82^. Deepening our understanding of this interspecific and intraspecific variation may provide opportunities to develop more targeted management programs specific to *D. suzukii*.

## Materials and Methods

### Flies

A laboratory reared colony of *D. suzukii* was established using adults and larvae collected from raspberry fields (Germantown and Woodbine, MD, USA) as well as adults trapped in a residential riparian area (Beltsville, MD, USA) in 2014. Flies were reared for over 50 generations under a 16:8 hour light/dark cycle at 22 °C on a modified Bloomington Drosophila Stock Center cornmeal, molassess, and yeast medium (consisting of 84.4% v/v water, 9.6% v/v cornmeal, 5.5% w/v yeast, 4.6% v/v molasses, 0.5% w/v agar, 0.5% w/v proprionic acid, and 0.01% w/v methyl 4-hydroxybenzoate). Our colony recipe contains a higher concentration of yeast compared to the Bloomington recipe and uses different antifungals (proprionic acid and methyl 4-hydroxybenzoate instead of p-hydroxybenzoic acid methyl ester). However, all other ingredients and ratios were similar (Supplemental Table S1). The colony was infected with an unknown insect pathogen, which presented symptoms similar to *Drosophila* C Virus^83^; infected larvae typically exited the food at an early instar and developed a brownish-black coloration before dying. To minimize effects from this infection, fly bottles were carefully inspected prior to experiment, with flies only taken from bottles that did not exhibit active symptoms. Because development studies were completed using amicrobial larvae, we anticipate no confounding effects due to this infection.

### Yeasts

Experiments were conducted using five different species of yeast. Four of those species, *Hanseniaspora uvarum*, *Pichia kluyveri*, *Issatchenkia terricola*, and *Wickerhamomyces pijperi*, were isolated from the fecal pools (frass) of field-collected *D. suzukii* larvae^38^ with individual yeast species selected based on the strength of their association with *D. suzukii*. In particular, *H. uvarum*, *P. kluyveri*, and *I. terricola*, were isolated from multiple populations of *D. suzukii* in both Maryland and California^38,39^. A strain of *Saccharomyces cerevisiae* obtained from Red Star^®^ Active Dry Yeast (LeSaffre Yeast Corporation, Milwaukee, WI, USA) was also included in laboratory assays as a positive control.

*H. uvarum*, *P. kluyveri*, *I. terricola*, and *S. cerevisiae* were used in all experiments described below, but *W. pijperi* was only included in the yeast preference assays. This species of yeast was only found in one field site in Maryland, but occured in 4 out of 12 larvae surveyed^38^. Given its strong prevelance at this single field site, we assayed larval yeast preference for *W. pijperi*. However, we excluded *W. pijperi* from the larval development assays due to labor constraints; larvae did not show a strong preference for that yeast and *W. pijperi* is not commonly associated with *Drosophila* spp.^39,47^.

### Yeast impacts on larval growth and development

To evaluate yeast impacts on larval fitness and development, *D. suzukii* larvae were reared on the same colony diet described above using a standardized quantity of one of four yeast species: *H. uvarum*, *P. kluyveri*, *I. terricola*, and *S. cerevisiae* (see diet recipe in Supplementary Table S1). As a negative control, diets were also prepared with no yeast added. All diets were steam sterilized using an autoclave at 121 °C for 20 minutes prior to use in experiments; this step killed all microbes and ensured that yeast quantity remained constant throughout the experiment. Approximately 18 grams of diet were poured into small (60 × 15 mm) petri dishes and cooled overnight in a sterile biosafety cabinet.

After the overnight cooling period, 20 amicrobial first-instar larvae were then added to each petri dish (Supplementary Methods). This created a density of 1.1 larvae g^−1^ diet, which is slightly below the threshold at which larval *D. suzukii* begin exhibiting significant competition effects^11^. The entire experiment was repeated on three separate dates (N = 3 replicates). During each experimental replicate, we prepared six subsamples per treatment (six petri dishes of diet containing 20 *D. suzukii* larvae each). *D. suzukii* larvae were monitored daily, and we quantified survivorship, development time, and adult body size (wing and thorax measurements) as measures of larval fitness.

Diet plates with larvae were held in a 22 °C incubator on a 16:8 hour light/dark cycle and checked daily at approximately the same time that each experimental replicate was initiated (generally between 10 AM and 12 PM). Any pupae that emerged were transferred from the diet into an individual 1.5 mL microcentrifuge tube and then monitored daily for adult emergence. Transferring the pupa into individual tubes ensured that the adult flies did not become stuck within the diet, thus allowing us to measure adult body size. Once emerged, adult flies were held in their tubes for 24 hours to harden before being frozen and stored for future body size measurements.

**Figure 5:**
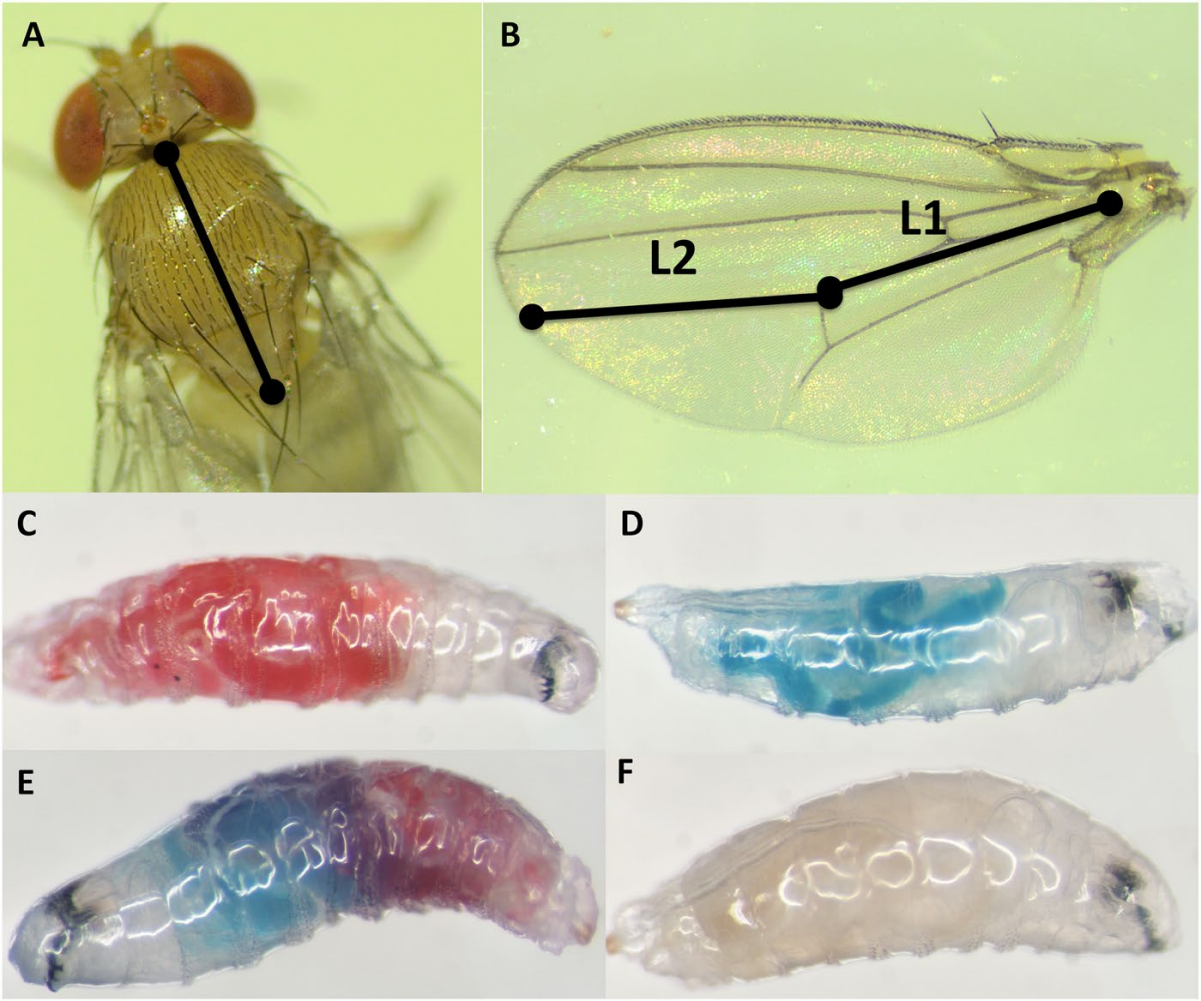
Methods for assessing larval development and yeast preferences. (**A**) Thorax length measurements were taken from the most anterior part of the mesothorax to the tip of the scutellum using a Leica M80 microscope with a reticle attached. (**B**) To quantify wing length, one wing was removed from each adult specimen and mounted on a glass slide. Two measurements were taken on each wing: L1 = the distance between the origin of the 4^th^ longitudinal vein to the posterior cross vein; L2 = the distance between posterior cross vein to the intersection of the wing edge and the longitudinal fourth vein. In larval yeast preference assay, larvae were scored as either (**C**) red, (**D**) blue, (**E**) purple, or (**F**) white.

To quantify adult body size, we measured the wing and thorax length for every adult *D. suzukii* that successfully emerged in our trials, using a Leica M80 microscope (Leica Microsystems, Wetzlar, Germany) with a reticle attached to the eyepiece. Measurements were adapted from previously described methods^84,85^. Briefly, to take thorax measurements, fine-tipped forceps were used to grasp each fly at the base of their legs, and the fly was oriented so its thorax was horizontal. Measurements were taken from the most anterior part of the mesothorax to the tip of the scutellum (Fig. 5A). Once thorax measurements were complete, the right wing was removed from the specimen and slide mounted. Two measurements were taken to quantify wing length: from the origin of the 4^th^ longitudinal vein to the posterior cross vein (L1; Fig. 5B) and from the posterior cross vein to the intersection of the wing edge and the 4^th^ longitudinal vein (L2; Fig. 5B). To minimize measurement biases and errors, all flies within a given replicate were measured by the same individual.

#### Statistical analysis

All statistical analysis were conducted using R.3.4.1^86^. Data were averaged across subsamples within an individual trial (N = 3 replicate trials). Survivorship rates were calculated as the percentage of larvae that sucessfully pupated (larval survivorship), the percentage of pupae that sucessfully emerged as adults (pupal survivorship), and the percentage of larvae that sucessfully emerged as adults (total survivorship). For each category, data were analyzed using a linear mixed model, with the percent survivorship as the response variable, yeast treatment included as a categorical predictor, and replicate included as a random effect. Model residuals were checked for the assumptions of normality of variance and homogeneity of variance using Shapiro-Wilk and Levene’s tests. In all three analysis, assumptions were satisfied using untransformed data. Significant results were followed by pairwise mean comparisons using Tukey’s adjustment in the lsmeans package^87^.

Development time to pupation (larval development), time from pupation to adult eclosion (pupal development), and total development (1^st^ instar larva to adult) were analyzed separately using a mixed-model ANOVA in the lme4^88^ and lmerTest^89^ packages in R, with yeast treatment included as a main effect and trial replicate as a random effect. We confirmed data met the assumption of normality of variance with Shapiro-Wilk tests. However, weighted least squares methods [weighting factor: (treatment residual variance)^−1^] were used for larval and total development times due to difficulties satisfying the assumption of homogeneous variance. No weighting factor was required for analysis of pupal development time.

Body size measurements were also analyzed using a mixed-model ANOVA using the lme4 and lmerTest packages and models again included the yeast treatment as a fixed effect and trial replicate as a random effect. Data were transformed as necessary to meet assumptions of normality and homogeneity of variance. Due to difficulties satisfying assumptions of normality and homogeneity of variances, the analyses were conducted separately for male and female flies, with female wing and thorax measurements analyzed using weighted least squares [weighting factor: (treatment residual variance)^−1^]. Significant results were again followed by pairwise mean comparisons using Tukey’s adjustment in the lsmeans package^87^.

### Quantifying diet quality

To compare the nutritional content of our experimental diets with the standard laboratory rearing diet, we conducted proximate nutrient analysis on each experimental diet as well as a diet prepared using freeze-dried *S. cerevisiae*. On two separate dates, diets were prepared using the same protocol described in 4.3. After autoclaving, all diets were poured into sterile 50 mL falcon tubes, refrigerated, and shipped to an off-site facility for analysis within three days of preparation. All analyses were conducted by Medallion Labs (General Mills D.B.A. Medallion Labs, Minneapolis, MN). Analysis were conducted 28 November 2018 and 9 January 2019 using standard testing protocols (Supplementary Methods).

### Evaluating larval yeast preference

Using binary choice feeding assays, we evaluated larval *D. suzukii*’s preference for five species of yeast (described above). Bioassay arenas were constructed following methods adapted from previous larval *Drosophila* feeding assays^32^ (Supplementary Methods). Briefly, in each experimental replicate, larvae were presented with two yeast species stained red and blue with food coloring. After one hour, larvae were removed and visually scored for yeast feeding preferences.

Second-instar *D. suzukii* larvae were starved for one hour prior to starting the assay (Supplementary Methods). Forty larvae were then transferred to the center of one assay arena using an ethanol sterilized paintbrush and left in dark conditions for one hour, during which time they were free to crawl around and feed on either yeast option. At the end of the hour assay period, larvae were individually removed from the arena, and scored for feeding preference using a Leica M80 stereomicroscope based on the color of their abdomen (Fig. 5C–F). Each larva could be classified as either red, blue, purple (indicating that they fed on both yeasts), or white (indicating that no choice was made).

#### Statistical Analysis

Any larvae that died or went missing during the hour-long assay period were excluded from the analysis. Prior to analysis, the number of larvae that chose to feed on each yeast option within an assay arena were standardized using a preference index described in Eq. (1) ^32^:

1 $${\rm{Larvae}}\,{\rm{with}}\,{\rm{colored}}\,({\rm{red}}\,{\rm{or}}\,{\rm{blue}})\,{\rm{abdomen}}+\frac{{\rm{Larvae}}\,{\rm{with}}\,{\rm{purple}}\,{\rm{abdomen}}}{2}$$

Adjusted larval counts were analyzed using a paired t-test^86^, with each assay arena of 40 larvae treated as an experimental replicate. Data were graphically checked for outliers using both box plots and Q-Q normality plots, and the assumption that the sampling distribution of mean differences was normally distributed was assessed using Anderson Darling test for normality in R with the ‘nortest’ package^90^. Data is reported as the percentage of larvae that chose to feed on each yeast.

#### Preference assay controls

To ensure that food coloring did not impact larval performance, we alternated which color each yeast option was stained between replicates. Additionally, a series of control preference assays was also conducted, in which larvae were presented with the same species of yeast in both food colors. Food coloring did not impact larval preference for any of the yeasts assayed (Supplementary Table S6).

To confirm that our visual assessments of larval feeding matched their actual feeding behavior, we also performed a set of separate confirmation assays and sequence identified the gut microbial community for a subset of experimental larvae (Supplementary Methods); results indicated that larval yeast feeding corresponded with the color of their abdomen, with few exceptions (Supplementary Table S7).

## Supplementary information

Supplementary Materials

## Data Availability

The datasets generated during the current study are available from the corresponding author upon reasonable request.
