# Supplementary material for: Differential Impacts of Yeasts on Feeding Behavior and Development in Larval Drosophila suzukii (Diptera:Drosophilidae)
Source: Sci Rep. 2019 Sep 16;9:13370. doi: 10.1038/s41598-019-48863-1 (PMC6746873; doi:10.1038/s41598-019-48863-1)
Supplement: Supplementary file 1 — Supplementary Materials [file 41598_2019_48863_MOESM1_ESM.pdf]

Differential Impacts of Yeasts on Feeding Behavior and Development in Larval *Drosophila suzukii* (Diptera:Drosophilidae)

Margaret T. Lewis* and Kelly A. Hamby

Department of Entomology, University of Maryland, College Park 20742

*mtlewis@umd.edu

Supplemental Methods

Assay Arena Construction and Setup

Assay arenas were constructed using large (100 × 15 mm) petri dishes filled with ~ 20 mL of 2.74% water agar (deionized water and granulated agar; Fischer BioReagents, Fairlawn, NJ, USA). Once the agar solidified, two 7 mm cores were removed from either end of the petri dish using the large end of a sterile 1000 uL pipette tip (Mettler Toledo Rainin Instrument, LLC, Oakland, CA, United States). The holes were filled with freshly autoclaved potato dextrose agar (PDA; Difco™; Sparks, MD, USA) and allowed to cool. Yeast options were plated on each PDA core and allowed to incubate at room temperature 48 hours prior to starting the assay. To visualize larval feeding preferences, 6 uL of autoclaved neon pink or neon blue food coloring (McCormick and Company, Inc.) were pipetted onto each core five minutes prior to starting each assay.

All preference assays were conducted using second instar *Drosophila suzukii* larvae that were approximately 48 hours old. To standardize the age of the larvae, female *D. suzukii* adults

were first placed on a grape juice agar plate for 48-72 hours, during which time they laid eggs within the media. Eggs were then collected using fine-tipped forceps, transferred to a small petri dish (60 × 15 mm) along with a small chunk of *Drosophila* diet, and incubated at 22°C for approximately 48 hours, at which point larvae were collected for assays.

To extract the larvae from the food, the diet was gently crushed and squirted with water, and larvae were removed using fine tipped paint brushes. All larvae were visually inspected under the microscope to confirm that they had reached the second instar by examining their mouth parts and the posterior/anterior spiracles. Briefly, first instar larva have very small mouthparts that appear to be small black dots, and the second instar larvae have larger and clearer mouth hooks. Second instar larvae also have a clubbed anterior spiracle, while third instar larvae have a fanned anterior spiracle and dark orange rings on their posterior spiracle (Shingleton Lab, Michigan State University, Discriminating Among *Drosophila* Instars).

We then transferred approximately 40 second instar larvae into a small petri dish containing a single moistened sheet of filter paper, and held them without food for one hour to increase the likelihood of a choice being made. After the starvation period, 40 larvae were transferred into the assay arena (each larva was visually inspected to ensure that it had not been damaged during the one hour starvation period).

Yeast Feeding Confirmation

To confirm that our visual metric (color) of larval *D. suzukii* preference reflected actual larval feeding, we evaluated the gut microbial community in confirmation assays that were conducted separately from larval yeast preference assays. Confirmation assays were only conducted using *H. uvarum*, *P. kluyveri*, and *S. cerevisiae*. Each combination of yeast

comparisons was replicated twice for these confirmation assays. Between replicates, we switched the color of food coloring (red or blue) used to stain each yeast species.

Larval preference assays were set up exactly as previously described for all yeast treatment combinations, and larvae were allowed one hour to feed. At the end of the hour feeding period, one larva typical of a red, blue, and purple specimen was removed from the assay arena, and the culturable gut microbial community was assessed (Hamby et al. 2012). Briefly, each larva was surface sterilized using 70% ethanol, placed in the center of a Rose Bengal chloramphenicol agar plate (RBCA) and allowed to crawl over the surface for 30-60 minutes. Plates were prepared using Rose Bengal chloramphenicol agar base (Oxoid, United Kingdom) according to the manufacturer's instructions and amended with 0.1 g/L chloramphenicol (Sigma-Aldrich, St. Louis, MO, USA). While on the media, each larva deposited fecal pools containing fungi that survived digestion. We isolated and purified two colonies of each yeast morphological type. One strain was then randomly selected to be sequence identified to the species or genus level using the methods described in Lewis et al. 2018. As an additional control, we also isolated the culturable gut microbial community from two larvae that were starved for the one hour period but did not feed on any yeast.

Overall, these confirmation assays indicate that larval *D. suzukii* fed on yeasts in the larval feeding preference assays. Our yeast feeding assessments lined up fairly well with the larval gut microbial community, particularly in comparisons between *H. uvarum* and *S. cerevisiae* (Table S7). In two replicate assays involving *Pichia kluyveri*, we did isolate *P. kluyveri* from larvae scored as having fed exclusively on the alternative yeast (denoted in bold on Table S7). It is possible that this discrepancy reflects imperfect surface sterilization of the larvae in ethanol; larvae may have picked up yeast spores while moving through the arena.

Alternatively, the food coloring that we used may not have stained *P. kluyveri* as well as the other yeast species; consequentially, larval preference for *P. kluyveri* may have been underestimated in these assays. We also found that the majority of larvae within this study had an additional yeast contaminant within their digestive tract, *Pichia occidentalis.* Given that we used non-sterile larvae in the preference assays, it seems likely that this yeast was present in our colony at the time of the experiments.

Nutritional Analysis

All nutritional analyses were performed by Medallion Labs (General Mills D.B.A. Medallion Labs, Minneapolis, MN, Analysis Conducted 28 November 2018 and 9 January 2019).

Protein (Dumas) Test

To determine total protein content a minimum of 20 grams of diet was combusted at a high temperature in the presence of pure O_2. Any non-nitrogenous combustion products (water vapor, oxygen, and CO_2) were removed with thermoelectic coolers and chemical sorbents, leaving only N_2 and NO_x. The latter product was reduced to N_2 by passage through a column packed with hot copper. The quantity of remaining N_2 was then determined and multiplied by a predetermined conversion factor (6.25), providing an estimate of total protein content as a percent of the original sample weight. This test had a detection limit of 0.125% nitrogen or 0.781% protein.

Fat by Gravimetric Test

Lipids were extracted from a minimum of 10 grams of fly diet via hydrolisis and partitionined into a mixed ether. The extract was dried and weighed, providing a measure of lipid

residue as a percent of the total original sample weight. This test had a detection limit of 0.1% lipid.

Ash Test

Total ash content was determined by combusting a minimum of 10 grams of fly diet at a high temperature. Once the remaining residue cooled, it was weighed and reported as a percentage of the original sample weight. This test provided a measure of all non-combustible minerals within the fly diet, including salts, carbonates, and silicates. The detection limit for this test was 0.012%.

Moisture Test

To determine the total moisture content, a minimum of 10 grams of fly diet were heated in an oven for a specified period of time. The remaining fly diet was then weighed, and the percent weight loss calculated as an estimate of moisture. This test had a detection limit of 0.012%.

Carbohydrates and Calories by Calculation

The total carbohydrate content (g/100g diet) and total calories were estimated using results from the ash, fat, moisture, and protein analysis. Total calories and carbohydrate were calculated using the following equations respectively : $Carbohydrates = 100 - (\%Ash) - (\%\ Total\ Fat) - (\%\ Moisture) - (\%\ Protein)$ and $Calories = (4\ \times\ Carbohydrates) + (9\ \times\ Total\ Fat) + (4\ \times\ Protein)$.

Supplemental Figures and Tables

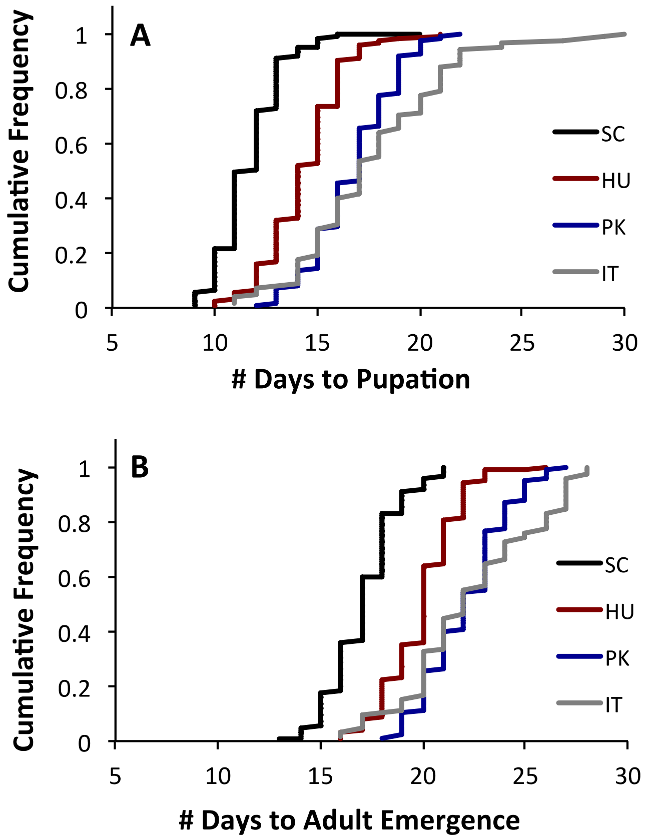

Figure S1. Cumulative frequency curves for the (A) larval (1st instar larvae to pupa) development time and (B) total (1st instar to adult) development time.

Table S1. Recipe used to prepare experimental diets for the larval development studies. Diets were prepared following the standard yeast-cornmeal-molasses diet used to maintain our laboratory stocks. Diets were prepared using no yeast (Control) or with a standardized quantity of frozen and heat-killed *H. uvarum* (HU), *P. kluyveri* (PK), *I. terricola* (IT), or *S. cerevisiae* (SC). To compare the nutritional value of these experimental diets with a standard *Drosophila* stock recipe (used to rear our laboratory colony and purchased from a University of Maryland facility for the colony), we conducted proximate nutritional analysis on all diets used in the development study as well as for the laboratory standard diet that was prepared using freeze-dried *S. cerevisiae*.

**Ingredient**	**Control**	**HU, SC, PK, or IT**	**Freeze-Dried SC***
Water	82.5 mL	82.5 mL	82.5 mL
Molasses	4.5 mL	4.5 mL	4.5 mL
Agar	0.468 g	0.468 g	0.468 g
Yeast	NA	5.4 g	5.4 g
Cornmeal	6.84 g	6.84 g	6.84 g
Tegosept	1.4 mL	1.4 mL	1.4 mL
Propionic acid	0.233 g	0.233 g	0.233 g

***** Diets were only prepared using freeze-dried *Saccharomyces cerevisiae* for use in proximate nutritional analysis

Table S2. Proximate nutrient analysis of experimental diets used in *D. suzukii* development studies. Diets were analyzed for total caloric content (presented as the calories per 100 grams) and the relative percentage of ash, moisture, carbohydrates, fats, and protein using standard proximate methods. All nutritional analysis were performed by Medallion Labs Inc. and repeated twice using diet prepared on two separate dates (Rep 1 and Rep 2). In some samples, the percentage of total fat and protein fell below the minimum detectable threshold and is indicated by the notations <0.5 and <0.781 respectively.

Treatment	Calories (per 100g)		Percent Ash		Percent Moisture		Percent Carb		Percent Fat		Percent Protein	
	**Rep 1**	**Rep 2**	**Rep 1**	**Rep 2**	**Rep 1**	**Rep 2**	**Rep 1**	**Rep 2**	**Rep 1**	**Rep 2**	**Rep 1**	**Rep 2**
CON	38	37	0.222	0.218	90.271	90.486	9.5	9.3	< 0.5	<0.5	< 0.781	<.781
HU	44	42	0.276	0.332	88.781	89.094	10.9	9.4	< 0.5	<0.5	< 0.781	1.16
IT	41	40	0.207	0.281	89.486	89.786	9.2	9.9	< 0.5	<0.5	1.12	<.781
PK	41	38	0.272	0.267	89.548	90.324	9.2	9.4	< 0.5	<0.5	1.01	<.781
SC	41	39	0.245	0.318	89.48	89.886	8.6	9.8	< 0.5	<0.5	1.69	<.781
DRY	52	53	0.385	0.462	86.653	86.191	10.4	11.1	< 0.5	<0.5	2.57	2.27

Table S3. Larval *D. suzukii* feeding preferences for all comparisons involving *Pichia kluyveri*. Data is presented as the mean percentage of larvae ± standard error that responded to each yeast (N=12 replicate binary choice assays). HU = *Hanseniaspora uvarum,* IT = *Issatchenkia terricola*, PK = *Pichia kluyveri*, SC = *Saccharomyces cerevisiae,* WP = *Wickerhamomyces pijperi*.

**Comparison**	**% PK**	**% Other**	***T***	**df**	***P*(T<0.05)**
PK - IT	34.8 ± 2.0	35.2 ± 3.1	0.114	11	0.912
PK - HU	27.1 ± 2.2	59.7 ± 2.5	7.468	11	< 0.001
PK - SC	33.7 ± 2.5	42.2 ± 2.6	-2.057	11	0.032
PK - WP	35.7 ± 2.4	37.2 ± 3.0	-0.516	11	0.616

Table S4. Larval *D. suzukii* feeding preferences for all comparisons involving *Issatchenkia terricola*. Data is presented as the mean percentage of larvae ± standard error that responded to each yeast (N=12 replicate binary choice assays). HU = *Hanseniaspora uvarum,* IT = *Issatchenkia terricola*, PK = *Pichia kluyveri*, SC = *Saccharomyces cerevisiae,* WP = *Wickerhamomyces pijperi*.

**Comparison**	**% IT**	**% Other**	**T**	**df**	***P*(T<0.05)**
IT - PK	35.2 ± 3.1	34.8 ± 2.0	0.114	11	0.912
IT - HU	23.1 ± 2.1	61.4 ± 2.6	8.601	11	< 0.001
IT - SC	31.5 ± 4.8	24.8 ± 3.7	-1.211	11	0.251
IT - WP	41.6 ± 3.8	32.7 ± 3.1	1.775	11	0.104

Table S5. Larval *D. suzukii* feeding preferences for all comparisons involving *Wickerhamomyces pijperi*. Data is presented as the mean percentage of larvae ± standard error that responded to each yeast (N=12 replicate binary choice assays). HU = *Hanseniaspora uvarum,* IT = *Issatchenkia terricola*, PK = *Pichia kluyveri*, SC = *Saccharomyces cerevisiae,* WP = *Wickerhamomyces pijperi*.

**Comparison**	**% WP**	**% Other**	***T***	**df**	***P*(T<0.05)**
WP - IT	32.7 ± 3.1	41.6 ± 3.8	1.775	11	0.104
WP - PK	37.2 ± 3.0	35.7 ± 2.4	-0.516	11	0.616
WP - HU	30.1 ± 3.9	50.9 ± 3.8	3.042	11	0.011
WP - SC	45.9 ± 4.3	28.6 ± 3.5	-2.286	11	0.043

Table S6. Larval feeding in control yeast preference assays. Data is presented as the mean percentage ± standard error of larval *D. suzukii* that chose to feed on either the red or blue yeast in control yeast preference assays. HU = *Hanseniaspora uvarum*, IT = *Issatchenkia terricola*, PK = *Pichia kluyveri*, SC = *Saccharomyces cerevisiae*, WP = *Wickerhamomyces pijperi*.

**Comparison**	**% Red Larvae**	**% Blue Larvae**	**% Non-Responders**	***T***	**df**	***P*(*T*<0.05)**
HU-HU	45.9 ± 4.2	43.1 ± 3.4	11.0 ± 2.3	0.644	5	0.5477
PK-PK	43.4 ± 4.0	43.7 ± 3.3	12.9 ± 3.9	-0.071	5	0.946
SC-SC	34.8 ± 5.3	36.6 ± 4.5	28.6 ± 9.5	-0.791	5	0.465
IT-IT	26.2 ± 2.1	29.2 ± 2.8	44.6 ± 3.3	-0.795	5	0.457
WP-WP	39.5 ± 5.2	37.7 ± 3.1	22.8 ± 6.9	0.474	5	0.656

Table S7. Molecular identification to confirm larval yeast feeding in laboratory preference assays. Yeast strains isolated from larvae that were scored as either red, blue, or purple in binary yeast preference assays. All yeast strains were isolated and sequence identified to species. Data for each strain identified is presented, including the percent homology, the number of base pairs in the sequence, and the GenBank match accession number. The percentage of total colonies belonging to each morphospecies was visually estimated. Strains in bold represent identifications that contradict our visual assessment of yeast feeding.

Comparison	Rep	Red Yeast	Blue Yeast	Specimen color	% Homology	Bp	GenBank Match Accession #	% of Colonies	Genus	Species
HU vs PK	1	HU	PK	Red	100	547	KT922472.1	100%	*Hanseniaspora*	*uvarum*
		HU	PK	Blue	100	526	U76348.1	100%	*Pichia*	*occidentalis*
		HU	PK	Purple	99.8	550	U75727.1	99%	*Pichia*	*kluyveri*
		HU	PK	Purple	100	551	DQ104733.1	1%	*Pichia*	*kluyveri*
	2	PK	HU	Red	100	537	AB847520.1	100%	*Pichia*	*occidentalis*
		PK	HU	Blue	100	548	KT922893.1	80%	*Hanseniaspora*	*uvarum*
		**PK**	**HU**	**Blue**	**99.8**	**538**	**U75727.1**	**10%**	***Pichia***	***kluyveri***
		PK	HU	Blue	100	542	KT9230371.1	10%	*Hanseniaspora*	*uvarum*
		PK	HU	Purple	99.8	532	DQ104733.1	70%	*Pichia*	*kluyveri*
		PK	HU	Purple	99.8	536	U75727.1	30%	*Pichia*	*kluyveri*
HU vs SC	1	HU	SC	Red	100	553	KT922893.1	99%	*Hanseniaspora*	*uvarum*
		HU	SC	Red	100	504	KT923037.1	1%	*Hanseniaspora*	*uvarum*
		HU	SC	Blue	100	541	HM165257.1	98%	*Saccharomyces*	*cerevisiae*
		HU	SC	Blue	100	535	U76348.1	2%	*Pichia*	*occidentalis*
		HU	SC	Purple	99.3	560	HM165257.1	95%	*Saccharomyces*	*cerevisiae*
		HU	SC	Purple	100	534	U76348.1	5%	*Pichia*	*occidentalis*
	2	SC	HU	Purple	100	552	GP011558.1	99%	*Saccharomyces*	*cerevisiae*
		SC	HU	Purple	100	549	CP011558.1	1%	*Saccharomyces*	*cerevisiae*
		SC	HU	Blue	99.8	549	KT923037.1	99%	*Hanseniaspora*	*uvarum*
		SC	HU	Blue	100	550	KT923037.1	1%	*Hanseniaspora*	*uvarum*
		SC	HU	Red	100	553	HM165257.1	100%	*Saccharomyces*	*cerevisiae*

Comparison	Rep	Red Yeast	Blue Yeast	Specimen color	% Homology	Bp	GenBank Match Accession #	% of Colonies	Genus	Species
SC vs PK	1*	PK	SC	Blue	100	552	HM165257.1	30%	*Saccharomyces*	*cerevisiae*
		**PK**	**SC**	**Blue**	**99.8**	**533**	**DQ104733.1**	**50%**	***Pichia***	***kluyveri***
		**PK**	**SC**	**Blue**	**99.8**	**537**	**U75727.1**	**20%**	***Pichia***	***kluyveri***
		PK	SC	Purple	100	542	DQ104733.1	50%	*Pichia*	*kluyveri*
		PK	SC	Purple	99.6	535	DQ104733.1	50%	*Pichia*	*kluyveri*
		PK	SC	Purple	99.6	546	U75727.1	<1%	*Pichia*	*kluyveri*
	2	SC	PK	Red	100	536	U76348.1	99%	*Pichia*	*occidentalis*
		SC	PK	Red	100	552	CP011558.1	1%	*Saccharomyces*	*cerevisiae*
		SC	PK	Blue	100	542	DQ104733.1	100%	*Pichia*	*kluyveri*
		SC	PK	Purple	99.8	524	AM397862.1	100%	*Pichia*	*kluyveri*
CON	1	CON	CON	CON	100	540	AB847520.1	100%	*Pichia*	*occidentalis*
	2	CON	CON	CON	100	537	AB847520.1	100%	*Pichia*	*occidentalis*

***** In replicate 1 of the SC vs PK comparison, we also isolated yeasts (1 morphospecies, 100% of colonies) from one larva scored as red (feeding on *P. kluyveri*). The resulting chromatogram from that particular strain was messy and prevented us from making a genus or species level identification. However, morphological descriptions suggest that the strain was more likely *Pichia* sp. rather than *Saccharomyces* sp.
